# Immediate postoperative course in the pediatric intensive care unit following epilepsy surgery

**DOI:** 10.1007/s00381-024-06681-5

**Published:** 2024-12-06

**Authors:** Itay Ayalon, Shirley Friedman, Barak Meidan, Efraim Sadot, Shlomi Constantini, Shimrit Uliel-Sibony, Jonathan Roth

**Affiliations:** 1 Pediatric Intensive Care Unit, affiliated to the Faculty of Medical and Health Sciences Tel Aviv University, Dana-Dwek Children’s Hospital, Tel Aviv Sourasky Medical Center, 6 Weizmann Street, 6423906 Tel Aviv, Israel; 2 Department of Pediatric Neurosurgery, The Pediatric Brain Center, affiliated to the Faculty of Medical and Health Sciences, Tel Aviv University, Dana-Dwek Children’s Hospital, Tel Aviv Sourasky Medical Center, Tel Aviv, Israel; 3 Pediatric Neurology Unit, The Pediatric Brain Center, affiliated to the Faculty of Medical and Health Sciences, Tel Aviv University, Dana-Dwek Children’s Hospital, Tel Aviv Sourasky Medical Center, Tel Aviv, Israel

**Keywords:** Postoperative course, Hemispherotomy, Corpus callosotomy, Lesionectomy

## Abstract

**Purpose:**

To describe the immediate postoperative PICU course and short-term outcomes of children undergoing various epilepsy surgeries.

**Methods:**

Single-center, retrospective observational study. All patients younger than 20 years of age who had been admitted to the PICU between 2018 and 2022 following epilepsy surgery were eligible for study entry.

**Results:**

Fifty-two children (median age 7.9 years) underwent epilepsy surgery during the study period (25 focal lesionectomies and lobectomies [FL], 10 corpus callosotomy [CC], and 17 hemispheric surgeries [HS]). The average number of preoperative antiseizure medications (ASM) was 3, and the average number of failed ASM was 6. Cortical dysplasia was the most frequent etiology (25%). Preoperative cognitive delay and motor deficits were reported in 38 (74%) and 26 (50%) patients, respectively. The median length of stay in the PICU was 1 day (5 for the HS group). No seizures occurred among 44 (85%) children during the first postoperative day nor during the entire hospital stay in 40 (77%) patients (20/25 [82%] in the FL group, 4/10 [40%] in the CC group, and 14/17 [82%] in the HS group). There were no status epilepticus events during the PICU stay. None of patients required hemodynamic support, and only 3(6%) needed respiratory support. Twenty-six patients (50%) had electrolyte abnormalities. Pain was mostly perceived as mild. Fever was present in 28 (54%) patients, most notably in the HS group (94%).

**Conclusion:**

Epilepsy surgery in children is associated with very limited immediate postoperative morbidity and low seizure burden, especially in the FL and HS groups.

## Introduction

Epilepsy affects from 0.5 to 1% of children and is considered to be one of the most frequent chronic neurological condition of childhood [1]. The mainstay of therapy for epilepsy is antiseizure medications (ASM). Despite the availability of a variety of ASM, about 30% of children with epilepsy meet the International League Against Epilepsy (ILAE) criteria of drug-resistant epilepsy (DRE) [2], defined as a failure of adequate and well-tolerated trials of 2 ASMs [3]. DRE is associated with an increased risk for morbidity and mortality, and severely affects the quality of life of patients and their families [4]. Epilepsy surgery (ES) has gained popularity and has shown significant advantages in terms of seizure control and improvement of quality of life compared to medical treatment in both children and adults with DRE [5–7]. Formal guidelines for referral and evaluation for epilepsy surgery have been set by the ILAE Pediatric Epilepsy Surgery Task Force [8, 9], and patient selection and comprehensive pre-surgical evaluation performed in specialized epilepsy centers were found to correlate with improved long-term outcomes [7, 10].

ES in children may be extensive and debilitating, involving lobar resection, multilobar resection, and hemispheric disconnections. ES may be associated with prolonged anesthesia, blood loss, need for blood products, hemodynamic instability, and need for cardiovascular support, especially in the early infant age group [11]. These sequelae can be translated to a potentially prolonged and complicated stay in the pediatric intensive care unit (PICU). The seizure burden in the perioperative period or whether the surgery itself has the potential to trigger and provoke seizures in children whose seizure control is already fragile has not been conclusively established. Current literature provides considerable information about good long-term outcomes of pediatric ES [6, 11–13], but very little is known about the immediate postoperative course, including seizure control and PICU course of children following ES. Our primary goal, therefore, was to describe the postoperative PICU course and short-term outcomes of children undergoing various types of epilepsy surgeries.

## Materials and methods

### Study design and population

This is a retrospective, single-center, observational study. Following institutional review board approval (TLV- 0313–22), we reviewed the medical records of all consecutive pediatric patients (age 0–20 years) that were admitted to the PICU following ES between January 2018 and December 2022. Relevant demographic and clinical data as well as data on the specific surgical procedure and immediate postoperative outcomes were retrieved and analyzed. The length of follow-up included the postoperative course in the PICU and the stay in the pediatric neurosurgical department until discharged home or to a rehabilitation center.

The surgical interventions were divided into 3 subgroups: resection of focal lesions (FL) including lesionectomies and lobectomies, corpus callosotomy (CC) surgeries, and hemispheric surgeries (HS) including functional hemispherectomies. Stereotactic procedures, such as stereo-electroencephalography (SEEG) and laser ablation procedures (laser interstitial thermal therapy) were excluded.

The collected data included demographics, epilepsy-related information (age at seizure onset, duration of epilepsy before surgery, type and frequency of seizures, prior treatments [drugs, ketogenic diet, vagal nerve stimulation (VNS) etc.]), epilepsy-related etiologies, comorbidities, surgery-related details (type of surgery, hemodynamic stability, blood product use, anesthesia, and surgery times), PICU course-related data (mortality, number, type and burden of seizures, provision of respiratory and hemodynamic support, electrolyte abnormalities, liver and renal dysfunction, blood product use, occurrence of fever and infections, pain management, and PICU length of stay).

### Goals and outcomes

The first goal of this study was the evaluation of immediate postoperative complications during patients PICU and pediatric neurosurgical ward stay until hospital discharge. These included mortality, need for hemodynamic intervention (2 or more fluid boluses or need for vasoactive medications), need for respiratory support, electrolyte imbalances, renal and liver function abnormalities, coagulopathy and need for blood products, degree of pain as measured by validated and age-related pain scales (Face, Legs, Activity, Cry, Consolability [FLACC] for children under 3 years of age, Wong-Baker Faces for children between 3 and 7 years of age, and numerical rating scale [NRS] for children older than 7 years of age) and its management, occurrence of fever, and postoperative infectious complications.

The second goal was to assess the burden of seizures during the immediate postoperative period which we did by measuring the number of seizures from the first postoperative day until hospital discharge. We calculated the percentage of patients with complete cessation of seizures during their PICU and hospital stay, and used a subjective assessment based upon parental and treating team judgment of postoperative seizure burden.

### Statistical analysis

The collected data were tabulated in an Excel spreadsheet. All analyses were conducted by using the IBM SPSS statistical program (IBM SPSS Statistics for Windows, Version 27.0. Armonk, NY: IBM Corp.). Patient and surgery characteristics as well as data relevant to PICU stay and laboratory tests are presented as numbers or frequencies for categorical variables or medians with interquartile range [IQR] for continuous variables, as appropriate. All continuous variables were tested for normality of distribution by using the Kolmogorov–Smirnov test. Different types of surgeries were compared by one-way ANOVA (followed by Scheffe for post hoc analysis) for normally distributed continuous variables or Kruskal–Wallis for non-normally distributed variables. Categorical variables were compared using Pearson chi-square or Fisher’s exact tests, as appropriate. A logistic binary regression model was constructed by using a stepwise elimination, retaining variables in the final model that were statistically significant (*p* < 0.05). Nagelkerke *R* square was used to assess how well the model explains the tested variable. A *p* value < 0.05 was considered significant in all comparative analyses.

## Results

### Demographics and patient profiles

A total of 52 children underwent surgery for epilepsy (25 FL, 10 CC, and 17 HS) during the study period. The characteristics of the study cohort are presented in Table 1. The patients’ median age at the time of surgery was 7.9 (range 4.7–12.3) years, and those in the HS group were younger than the others, *p* = 0.07. The male-to-female ratio was roughly 3:1. There was a family history of epilepsy in 9 (17%) patients. The age of epilepsy onset was 1.5 (0.6–6.3) years, with no significant differences between groups. The duration of epilepsy before surgery was 4 (2.5–7.4) years. Seizure frequency prior to surgery was daily in 45 (88%) patients (100% among those in the CC and HS groups). Eleven (22%) patients had a history of status epilepticus. The median number of ASM at time of surgery was 3 (2–4), and the number of failed ASM was 6 (4–8) (eight ASM in the CC group, *p* = 0.03). Cortical dysplasia was the most frequent etiology of epilepsy, having been found in 13 (25%) of the patients in our total cohort (8 [32%] in FL and 5 [29%] in HS. Pathology was not sent for the CC group). A total of 17 of our patients had history of steroid treatment for epilepsy, 15 had followed a ketogenic diet, and 14 had VNS. Past VNS use was significantly more frequent among the patients in the CC group compared to the other 2 groups (*p* = 0.02). Cognitive delay was reported in 38 (74%) patients, with a significant difference between groups (50% in FL, 100% in CC, and 94% in HS, *p* < 0.001). A motor deficit was reported before surgery in 26 patients (20% in FL, 50% in CC, and 94% in HS, *p* < 0.001). Eight (15%) patients had a history of a neurological comorbidity.

**Table 1:** Patient characteristics

Characteristic	All patients *n* = 52	Focal resection *n* = 25 (48%)	Corpus callosotomy *n* = 10 (19%)	Hemispherotomy *n* = 17 (33%)	*p* value
Age – yrs [IQR]^a^	7.9 [4.7–12.3]	8.5 [5.2–13.8]	9.5 [7.7–14.4]	4.8 [3.9–7.9]	0.07^d^
Male sex (%)	73	72	90	65	0.35^e^
History of prematurity (%)	4	5	0	7.1	0.7^e^
Family history of epilepsy (%)	17	18	30	7	0.3^e^
Age of onset of epilepsy – yrs [IQR]^b^	1.5 [0.6–6.3]	3.5 [0.7–7.5]	1.5 [0.7–4.9]	1 [0.5–1.5]	0.2^c^
Duration of epilepsy until surgery– yrs [IQR]^b^	4 [2.5–7.4]	4.1[1–8.5]	7.2 [5.3–10.9]	3.7 [2.9–5.6]	0.7^c^
Frequency of seizures before surgery:					
Daily (%)	88	75	100	100	0.12^e^
Weekly (%)	10	21	0	0	
Monthly (%)	2	4	0	0	
History of status epilepticus (%)	22	18	50	13	0.06^e^
Number of previous ASM [IQR]^a^	6 [4–8]	6 [1–8]	8 [5–13]	4.5 [4–7]	***0.03***^d^
Number of current ASM [IQR]^b^	3 [2–4]	3 [2, 3]	3 [3, 4]	3 [2–4]	0.39^c^
Epilepsy-related etiology: Cortical dysplasia (%) Rasmussen encephalitis (%) Tuberos sclerosis (%) Post-trauma (%) Brain malignancy (%) Post-stroke (%) Hemimegalencephaly (%) Post-infection (%) Other diagnoses related to epilepsy (%)	25 6 4 4 15 11 4 8 22	32 4 8 4 32 0 0 4 4	n.a 0 0 0 0 0 0 20 70	29 12 0 6 0 35 12 6 24	0.13^e^ 0.39^e^ 0.33^e^ 0.75^e^ 0.06^e^ ** < *0.001***^e^ 0.12^e^ 0.26^e^ ** < *0.001***^*e*^
History of: IVIG treatment (%) Steroid treatment (%) Plasmapheresis treatment (%) Ketogenic diet (%) Vagal nerve stimulator (%) Previous neurosurgical intervention (%)	14 32 4 30 27 8	4 31 0 33 12 8	30 50 0 50 70 0	20 38 13 13 24 13	0.11^e^ 0.21^e^ 0.09^e^ 0.11^e^ ***0.02***^*e*^ 0.49^e^
Neurological examination pre-surgery Intellectual disability (%) Motor disability (%) Speech disability (%)	64 50 50	46 20 33	100 50 80	70 94 56	***0.01***^e^ ** < *0.001***^e^ ***0.04***^e^
Developmental delay (%)	74	50	100	94	** < *0.001***^e^
Total number of comorbidities [IQR]^b^	0 [1]	0 [0]	0 [1]	0 [1.5]	***0.05***^c^

^a^Normally distributed, ^b^not normally distributed, ^c^Kruskal-Wallis, ^d^one-way ANOVA + Scheffe, ^e^Pearson chi-square

*n.a.*, not applicable

### Surgery and course

Our study cohort underwent a total of 52 epilepsy surgeries (Table 2). The median time of surgery was 3 (2.4–3.9) hours (slightly longer in the FL group). Electrocorticography was used only in the FL group in 21 of the 25 surgeries. Hemodynamic instability (the provision of 2 or more fluid boluses or need for vasoactive medication) during surgery was recorded in only 3 (6%) cases. Intraoperative need for blood products was recorded in 8 (15%) surgeries (7 patients received packed red blood cells [PRBC] and 2 patients were given fresh-frozen plasma). An external ventricular drain (EVD) was used in all 17 HS cases, in 1 FL case, and in no CC cases.

**Table 2:** Surgery characteristics

Characteristic	All patients *n* = 52	Focal resection *n* = 25	Corpus callosotomy *n* = 10	Hemispherotomy *n* = 17	*p* value
Use of electrocorticography (ECOG) during surgery (%)	40	84	0	0	** < *0.001***^e^
Ventriculostomy placement during surgery (%)	35	4	0	100	** < *0.001***^e^
Blood products during surgery					
Any	15	16	0	24	0.26^e^
PRBC (%)	14	17	0	18	0.37^e^
PLT (%)	0	0	0	0	n.a
FFP (%)	4	4	0	6	0.74^e^
Cryoprecipitate (%)	0	0	0	0	n.a
Hemodynamic instability during surgery (%)	6	4	0	12	0.39
Anesthesia time (hours) [IQR]^a^	4.45 [3.4–5.5]	5 [4.4–5.8]	3.2 [3–3.9]	3.9 [2.9–5.6]	** < *0.001***^d^

^a^Normally distributed, ^c^Kruskal-Wallis, ^d^one-way ANOVA + Scheffe, ^e^Pearson chi-square

*n.a.*, not applicable

### Postoperative PICU course and immediateoutcomes (Tables 3 and 4)

**Table 3:** ICU course characteristics

Characteristic	All patients *n* = 52	Focal resection *n* = 25	Corpus callosotomy *n* = 10	Hemispherotomy *n* = 17	*p* value
ICU mortality (%)	0	0	0	0	
ICU length of stay (days)^b^ [IQR]	1 [1–5]	1 [1–1]	1 [1, 2]	5 [5–7]	** < ** ***0.001*** ^c^
Need for hemodynamic support during ICU stay (%)	0	0	0	0	
Need for respiratory support during 1st 24-h ICU stay (%)					
• Any support (%)	5.8	8	0	5.9	0.7^e^
• Oxygen (%)	5.8	8	0	5.9	0.7^e^
• Non-invasive (%) ventilation	0	0	0	0	0.6^e^
• Invasive ventilation (%)	2	4	0	0	
Electrolyte abnormalities during ICU stay					
• Any (%)	50	44	40	65	0.3^e^
• Sodium abnormality (%)	14	8	10	24	0.3^e^
• Potassium abnormality (%)	23	16	20	35	0.3^e^
• Other (%)	28	33	11	29	0.4^e^
Renal dysfunction (KDIGO^f^) (%)	0	0	0	0	
Abnormal liver transaminase levels (%)	8	12	0	6	0.5^e^
Need for blood products during ICU stay					
• Any (%)	4	8	0	0	0.3^e^
• PRBC (%)	4	8	0	0	0.3^e^
• PLT (%)	0	0	0	0	
• FFP (%)	0	0	0	0	
• Cryoprecipitate (%)	0	0	0	0	
Fever during ICU stay					** < ** ***0.001*** ^e^
• Any (%)	54	40	20	94	
• 38–39 °C (%)	21	20	20	24	
• > 39 °C (%)	33	20	0	70	
Fever starting day (postoperation day [IQR])^b^	1 [1, 2]	1 [1, 2]	2 [2–2]	1 [1, 2]	0.2^c^
Fever duration (days [IQR])^b^	4 [1–8]	1 [3, 4]	1.5 [n.a.]	7.5 [4–11]	***0.04*** ^c^
Positive cultures post-surgery (all patients)					
• Any	8 (*n* = 4)	4	10	12	0.6^e^
• Blood (%)	2 (*n* = 1)	0	10	0	0.1^e^
• Urine (%)	2 (*n* = 1)	0	0	6	0.3^e^
• CSF (%)	2 (*n* = 1)	0	0	6	0.3^e^
• Other (%)^k^	2 (*n* = 1)	4	0	0	0.6^e^
Worst pain intensity (1st 24 h)					
• Mild (%)	57	50	70	59	
• Moderate (%)	31	29	30	35	0.4^e^
• Severe (%)	12	21	0	6	
Opiate use (1st 24 h) (%)	17	16	10	24	0.6^e^

^a^Normally distributed, ^b^not normally distributed, ^c^Kruskal-Wallis, ^d^one-way ANOVA + Scheffe, ^e^Pearson chi-square, *n.a.*, not applicable, ^f^*KDIGO*, Kidney Disease Improving Global Outcomes, ^k^tracheal aspirate

**Table 4:** Laboratory characteristics during ICU stay

Characteristic	All patients *n* = 52	Focal resection *n* = 25	Corpus callosotomy *n* = 10	Hemispherotomy *n* = 17	*p* value
Hemoglobin change during ICU stay (g/dL) [IQR]^a^	− 1 [− 0.8 ( −) − 1.8]	− 0.9 [− 0.7 ( −) − 1.5]	− 0.8 [− 0.2 ( −) − 1]	− 1.8 [− 1.15 ( −) − 2.7]	***0.02*** ^d^
Maximal WBC count during ICU stay (all pt.) (10e3/μL) [IQR]^a^	13.9 [11.8–17.8]	13.6 [10–16.9]	13.9 [11.7–16.4]	15.4 [14.3–20.4]	0.06^d^
Maximal CRP during ICU stay (all patients) (mg/L) [IQR]^b^	59 [13–146]	65 [17–131]	12 [4–16]	148 [35–169]	***0.002*** ^c^
Maximal CRP day (all patients) (day) [IQR]^b^	1 [1, 2]	1 [1, 2]	1 [1–1]	2 [2–2]	***0.01*** ^c^
Minimal sodium level during ICU stay (mmol/L) [IQR]^a^	138 [136–139]	138 [137–141]	138 [137–139]	136 [134–137]	

*CRP*, C-reactive protein

^a^Normally distributed, ^b^not normally distributed, ^c^Kruskal-Wallis, ^d^one-way ANOVA + Scheffe

#### General

The median length of stay in the PICU was 1 [1-5] day (significantly longer for the HS group (5 [5-7] days) than the FL group (1 [1-1] day) and CC group (1 [1-2] day), *p* < 0.001), mostly related to the presence of an EVD (the median EVD duration was 5 days) which mandates an ICU stay by our hospital policy. There were no mortalities among our study participants.

#### Early postoperative seizure burden (Tables 5 and 6)

**Table 5:** Immediate post-surgery seizure burden

Variable	All patients *n* = 52	Focal resection *n* = 25	Corpus callosotomy *n* = 10	Hemispherotomy *n* = 17	*p* value
Patients without seizures during hospital stay (%)	77	82	40	82	0.11^e^
Patients without seizures during 1st 24 h postoperation (%)	85	96	60	82	***0.03*** ^***e***^
Patients without seizures following 24 h postoperation (%)	80	88	40	82	0.06^e^
Seizure burden compared to baseline^h^					0.14^e^
• Increased (%)	0	0	0	0	
• Decreased (%)	98	100	90	100	
• No change (%)	2	0	10	0	
Need for new antiepileptic drug or increasing pre-surgery doses (%)	6	4	10	6	0.79$
Status epilepticus during ICU course (%)	0	0	0	0	n.a
Need for EEG during ICU stay	0	0	0	0	n.a

^a^Normally distributed, ^b^not normally distributed, ^c^Kruskal-Wallis, ^d^one-way ANOVA + Scheffe, ^e^Pearson chi-square, ^h^based upon patient’s family perception

*n.a.*, not applicable

**Table 6:** Predictors seizures in the immediate post-surgery period

Variable	Univariate model		
	Seizures *n* = 9	No seizures *n* = 30	Odds ratio [95% CI]
Male (%)	73	74	0.8 [0.2–3.8]*
Age [IQR]^a^	7.9 [3.8–9.1]	7.9 [4.4–12.2]	01 [0.9–1.1]*
History of developmental delay (%)	68	100	n.a
Duration of epilepsy (yrs)^b^	7.1 [2.7–8.1]	3.9 [1.8–6.3]	1.1 [0.9–1.3]*
Number of current ASM^b^	3 [3, 4]	3 [2, 3]	1.8 [0.9–3.5]*
Number of previous ASM^a^	5 [4–13]	6 [1–7]	1.1 [0.89–1.3]*
Post-surgery fever (%)	50	55	0.8 [0.2–3.3]*
Post-surgery infection (%)	20	5	5 [0.6–41]*
EVD placement (%)	30	36	0.8 [0.2–3.4]*
Steroid treatment (%)	40	52	0.6 [0.1–2.5]*
Post-surgery electrolyte abnormality (%)	60	47	1.7 [0.4–6.7]*
Type of surgery			
• Focal resection (%)	20	55	0.2 [0.04–1.1]*
Corpus callosotomy (%)	50	12	7.4 [1.6–35]*, p ** = ** ***0.1***
• Hemispherotomy (%)	30	33	0.9 [0.2–3.8]*

^a^Normally distributed, ^b^not normally distributed

*n.a.*, not applicable

^*^Binary logistic regression—univariate, Nagelkerke *R* square – 0.19

Forty (77%) patients did not sustain any clinical seizures during their PICU and hospital stay (82% among FL and HS and only 40% among CC). No seizures occurred in the first postoperative day among 44 (85%) of all patients (96% in FL and 82% in HS compared to 60% in CC, *p* = 0.03). Following the first day of admission, 42 patients (80%) had no seizures by the time of discharge, with a trend towards significance between groups (88% in FL and 82% in HS compared to 40% in CC, *p* = 0.06). The patients’ primary caregivers described a significant decrease in seizure burden (number, duration, or intensity of seizures) during hospital stay in all but 1 CC patient whose primary caregiver indicated no change in seizure burden. ASMs were continued as prescribed before surgery in all cases and changed during hospital stay in only 3 (6%) cases. There were no occurrences of postoperative status epilepticus. We sought to explore potential predictors for immediate post-surgical seizures by using univariate and multivariate models (Table 6). The only variable that was found to be associated with immediate postoperative seizures was CC surgery (odds ratio [OR] 7.4). Of note, that variable explains only 20% (Nagelkereke R square – 0.19) of the occurrence of postoperative seizures. Since only 1 variable was found to be associated to seizures, the use of the multivariate model was not relevant.

#### Hemodynamic and respiratory support

None of the patients required hemodynamic support (2 or more fluid boluses or need for vasoactive medications) during their PICU stay. Only 3 (6%) of the patients needed any kind of respiratory support (2 patients needed oxygen supplementation through a nasal cannula and one patient needed invasive respiratory support). The only patient who needed invasive respiratory support was a 1-month-old who presented with refractory status epilepticus for which invasive mechanical ventilation was needed before surgical intervention. He subsequently underwent surgery for cortical dysplasia resection.

#### Electrolyte, renal, and liver abnormalities

Twenty-six patients (50%) had electrolyte abnormalities, with no difference between the 3 groups. The specific abnormalities included potassium (12 cases [23%]), hypomagnesemia (10 cases [19%]), hyponatremia (7 cases [13%], of which 6 cases were mild [mean sodium 132 meq/L] that resolved spontaneously, and 1 case that was moderate [128 meq/L] and treated as cerebral salt wasting), and phosphate (5 cases [10%]). Most of the abnormalities were self-limiting and needed no interventions other than follow-up until normalization. There were no cases of postoperative acute kidney injury. Four (8%) of all patients had mild reversible liver enzyme abnormalities.

#### Coagulopathy and need for blood products

Two patients needed blood products during their PICU stay (PRBC only). The median drop in patients’ hemoglobin during their PICU stay was 1 (0.8–1.8) gr/dL (higher [1.8 gr/dL] for the HS group, *p* = 0.02 compared to the other 2 groups). There was no case of severe bleeding or coagulopathy.

#### Pain management

During the first postoperative day, most patients (*n* = 29 [57%]) sustained mild pain, and 9 (17%) were treated with opioid drugs (no difference between groups). Twenty-six patients remained in the PICU during the second postoperative day (mainly HS patients), of whom 15 (60%) reported mild pain and 5 (19%) needed opioid drugs for pain relief. Twenty-two patients remained in the PICU during the third postoperative day, among whom 17 (77%) reported mild pain and 4 (18%) needed opioid drugs.

#### Fever and infectious complications

Twenty-eight (54%) patients had fever > 38 °C at any time during their entire hospitalization, and the fever of 17 of them was > 39 °C. The fever started on the first postoperative day and lasted 4 (1–8) days. There was a significant difference between the surgical groups: fever was very common in the HS group (94%, *p* < 0.001 compared to the other 2 groups) and lasted 7.5 days (*p* = 0.04). It was not found to be related to any infectious etiology. We had 1 case each of bacteremia (*Staphylococcus epidermidis*), meningitis (*Staphylococcus hominis*), urinary tract infection (*Escherichia coli*), and (*Staphylococcus aureus*). The infections were treated with antibiotics and had no long-term sequelae. Predictors for fever following surgery are presented in Table 7. A univariate model found a correlation between the occurrence of fever and type of surgery: HS patients had an increased risk with an OR of 31 (95% confidence interval 3–260), and CC patients had a decreased risk with an OR of 0.15 (0.1–0.8). Variables that were associated with increased risk for fever included a hemoglobin drop, a higher maximal white blood cell count, and a higher maximal C-reactive protein. Variables associated with a decreased risk for fever included perioperative steroid use and older age. A multivariate logistic analysis found the type of surgery as being the most important predictor of fever, with the HS patients having an OR of 22 [2.4–210], *p* = 0.01 compared to the other 2 groups. Of note, positive bacterial culture (any) was not found to be correlated with the occurrence of fever in our model.

**Table 7:** Predictors of fever following surgery

Variable	Univariate model			Multivariate logistic regression analysis odds ratio [95% CI]
	Fever *n* = 28	No fever *n* = 24	Odds ratio [95% CI]	
Male (%)	71	75	0.8 [0.2–2.9]*	
Age [IQR]^a^	6.2 [6]	9.3 [8]	0.9 [0.8–0.97]*	n.s
Steroid Tx (%)	36	67	0.28 [0.1–0.9]*	n.s
Hemoglobin drop during ICU stay gr/dL [IQR]^a^	0.9 [1]	1.5 [2]	3.1 [1.3–7.5]*	n.s
Hemoglobin drop (total) stay gr/dL [IQR]^a^	3.1 [2]	2.3 [2]	2.8 [1.4–5.4]*	Collinearity
Positive bacterial culture (%)	14	0	n.a	
Maximal white cell count during ICU stay (10e3/μL) [IQR]^a^	15.9 [6]	12.9 [6]	1.2 [1.1–1.4]*	n.s
Maximal CRP level during ICU stay (mg/L) [IQR]^b^	100 [113]	16 [20]	1.01 [1.01–1.025]*	1.01 [1–1.02] **p = 0.05
EVD placement (%)	60	4	35 [4–302]*	Collinearity
Type of surgery				
• Focal resection (%)	36	63	0.3 [0.1–1.1]	
• Corpus callosotomy (%)	7	34	0.15 [0.1–0.8]*	n.s,
• Hemispherotomy (%)	57	4	31 [3–260]*	22 [2.4–210] ** p = *0.01*

*CRP* C-reactive protein

^a^Normally distribution

^b^Not normally distributed

*n.a.* not applicable

*n.s.* not significant

^*^ Binary logistic regression—univariate

^**^Binary logistic regression—multivariate (forward stepwise conditional), Nagelkerke *R* square – 0.53

Collinearity between hemispherotomy and EVD placement

Collinearity between total hemoglobin drop and hemoglobin drop during ICU stay

#### Readmission and discharge

None of the patients were readmitted to the PICU and none needed additional surgical intervention during the same hospital stay. Four patients were readmitted to the hospital within 3 months after surgery: 1 each due to surgical site abscess that needed surgical drainage 2 months after FL resection, status epilepticus a few days following discharge after FL resection (only medical treatment was needed), or irritability related to bilateral subdural hygromas 1 week following FL resection (no surgical intervention was needed). One patient was admitted for evaluation of prolonged fever 1 month following HS (no infectious etiology was found, and the fever resolved).

A total of 13 out of 52 patients (25%) were discharged to rehabilitation centers (9/17 [53%] HS, 1/10 [10%] CC, 3/25 [12%] FL); the rest of the patients were discharged to their homes.

## Discussion

Increased awareness accompanied by advancements in technology and research have led to more widespread performance of ES worldwide. ES has clear long-term advantages over medical treatment for children with DRE in terms of seizure control rates, quality of life, and neurocognitive development. Surgery often includes extensive resections and disconnections and is often performed at an early age, which may pose a potential risk of sequelae beyond those associated with the surgery itself. In addition, these children are treated with various ASM which may potentially affect the immediate postoperative course. While the long-term outcomes of pediatric patients undergoing surgery for epilepsy has been studied in depth [6, 14, 15], our literature search yielded no reports on immediate or short-term outcomes nor any descriptions of their postoperative PICU course after they leave the operating theater. These topics are the focus of this study.

Our results suggest that the immediate postoperative course (including the PICU stay) is uneventful in most cases, that patients are not prone to seizure exacerbation, and that the seizure burden is significantly decreased. Patients undergoing FL or callosotomy had a median PICU stay of 1 day, while those undergoing HS had a median stay of 5 days (related to the presence of an EVD, which mandates ICU care in our institution). Many of the HS patients had high fever, mostly non-infectious. This has been described for HS and reportedly bears no long-term sequelae [16]. Other common findings included pain, mostly reported as being mild, with about 20% needing opioid treatment, as well as electrolyte imbalance, which was mostly mild and self-limiting. The occurrences of cerebral salt wasting and syndrome of inappropriate antidiuretic hormone secretion were rare (1/52 cases). Coagulopathy and the need for blood products were rare, even though 8 children had received blood transfusions intraoperatively. We also report a very low rate of the need for any respiratory (3 cases, 6%) or hemodynamic support (none).

The seizure burden in the immediate postoperative period of all 3 groups was significantly reduced compared to baseline, with 82% of patients in the FL and HS groups being seizure free during their hospital stay. Of note, most of those patients had sustained daily seizures prior to their surgery. In the CC group, as expected from the palliative nature of the procedure [17], most patients continued to experience seizures during their hospital stay (60%) but their seizure semiology, duration, and frequency changed and their seizure intensity was reduced according to their caregivers. Only a few of the patients needed to increase their ASM regimen (4% in the FL group and 6% in the HS group) and none of the patients had status epilepticus during their hospital stay.

Dwivedi et al. reported a freedom from seizures at 12 months in 77% of 57 pediatric patients that underwent epilepsy surgery (36 focal resections, 15 hemispherotomies, and 10 corpus callosotomies) [6]. Zhang et al. reported freedom from disabling seizures (Engel class 1) in 74% of children at least 12 months after they underwent focal cortical dysplasia resection [13]. Hoppe et al.’s questionnaire study [12] evaluated long-term (> 10 years) outcomes in subjects that underwent epilepsy surgery as children. Two-thirds of the responders was seizure free (Engel class 1). Of note, most of the patients who were seizure free at 1 year post-surgery were also seizure free at the last follow-up. In a recent meta-analysis that included 25 pediatric studies, Harris et al. reported a 1- and 10-year freedom from seizures in 74% and 61% of patients, respectively [18]. In our study, 77% of all patients were seizure free during their hospital stay, possibly implying that the immediate postoperative period may reflect the long-term highly beneficial effect of surgery.

### Study limitations

The main limitations of our study are its retrospective design and that it is based upon a review of medical charts that may subject our findings to information bias. Also, the relatively small sample size and the exceptionally high level of expertise of our neurosurgery department may limit generalization of our findings.

## Conclusion

Epilepsy surgery in children is associated with low immediate postoperative morbidity. The seizure burden in the immediate postoperative PICU course of patients undergoing epilepsy surgery is extremely low, especially in the FL and HS groups.

## Data Availability

The data that support the findings of this study are available from the corresponding author upon reasonable request.

## References

[1] Aaberg KM, Gunnes N, Bakken IJ, et al (2017) Incidence and prevalence of childhood epilepsy: a nationwide cohort study. Pediatrics 139. 10.1542/peds.2016-3908 10.1542/peds.2016-3908 28557750

[2] Kalilani L, Sun X, Pelgrims B et al (2018) The epidemiology of drug-resistant epilepsy: a systematic review and meta-analysis. Epilepsia 59:2179–2193. 10.1111/epi.14596 30426482 10.1111/epi.14596

[3] Kwan P, Arzimanoglou A, Berg AT et al (2009) Definition of drug resistant epilepsy: consensus proposal by the ad hoc Task Force of the ILAE Commission on Therapeutic Strategies: Definition of Drug Resistant Epilepsy. Epilepsia 51:1069–1077. 10.1111/j.1528-1167.2009.02397.x 19889013 10.1111/j.1528-1167.2009.02397.x

[4] Quintas R, Alvarez AS, Koutsogeorgou E et al (2012) The relationship between health-related quality-of-life and disability in patients with controlled epilepsy: a cross-sectional observational study. Am J Phys Med Rehabil 91:S31-38. 10.1097/PHM.0b013e31823d4db9 22193308 10.1097/PHM.0b013e31823d4db9

[5] Engel J, McDermott MP, Wiebe S et al (2012) Early surgical therapy for drug-resistant temporal lobe epilepsy: a randomized trial. JAMA 307:922–930. 10.1001/jama.2012.220 22396514 10.1001/jama.2012.220 PMC4821633

[6] Dwivedi R, Ramanujam B, Chandra PS et al (2017) Surgery for drug-resistant epilepsy in children. N Engl J Med 377:1639–1647. 10.1056/NEJMoa1615335 29069568 10.1056/NEJMoa1615335

[7] Ryvlin P, Cross JH, Rheims S (2014) Epilepsy surgery in children and adults. Lancet Neurol 13:1114–1126. 10.1016/S1474-4422(14)70156-5 25316018 10.1016/S1474-4422(14)70156-5

[8] Cross JH, Jayakar P, Nordli D et al (2006) Proposed criteria for referral and evaluation of children for epilepsy surgery: recommendations of the subcommission for pediatric epilepsy surgery. Epilepsia 47:952–959. 10.1111/j.1528-1167.2006.00569.x 16822241 10.1111/j.1528-1167.2006.00569.x

[9] Jehi L, Jette N, Kwon C-S et al (2022) Timing of referral to evaluate for epilepsy surgery: expert consensus recommendations from the Surgical Therapies Commission of the International League Against Epilepsy. Epilepsia 63:2491–2506. 10.1111/epi.17350 35842919 10.1111/epi.17350 PMC9562030

[10] Thijs RD, Surges R, O’Brien TJ, Sander JW (2019) Epilepsy in adults. Lancet 393:689–701. 10.1016/S0140-6736(18)32596-0 30686584 10.1016/S0140-6736(18)32596-0

[11] Roth J, Constantini S, Ekstein M et al (2021) Epilepsy surgery in infants up to 3 months of age: Safety, feasibility, and outcomes: a multicenter, multinational study. Epilepsia 62:1897–1906. 10.1111/epi.16959 34128544 10.1111/epi.16959

[12] Hoppe C, Beeres K, Witt J et al (2023) Clinical adult outcome 11–30 years after pediatric epilepsy surgery: complications and other surgical adverse events, seizure control, and cure of epilepsy. Epilepsia 64:335–347. 10.1111/epi.17477 36468792 10.1111/epi.17477

[13] Zhang S, Luo Y, Zhao Y et al (2023) Prognostic analysis in children with focal cortical dysplasia II undergoing epilepsy surgery: clinical and radiological factors. Front Neurol 14:1123429. 10.3389/fneur.2023.1123429 36949857 10.3389/fneur.2023.1123429 PMC10025379

[14] Téllez-Zenteno JF, Ronquillo LH, Moien-Afshari F, Wiebe S (2010) Surgical outcomes in lesional and non-lesional epilepsy: a systematic review and meta-analysis. Epilepsy Res 89:310–318. 10.1016/j.eplepsyres.2010.02.007 20227852 10.1016/j.eplepsyres.2010.02.007

[15] Dagar A, Chandra PS, Chaudhary K et al (2011) Epilepsy surgery in a pediatric population: a retrospective study of 129 children from a tertiary care hospital in a developing country along with assessment of quality of life. Pediatr Neurosurg 47:186–193. 10.1159/000334257 22213776 10.1159/000334257

[16] Kamath AA, Limbrick DL, Smyth MD (2015) Characterization of postoperative fevers after hemispherotomy. Childs Nerv Syst 31:291–296. 10.1007/s00381-014-2572-7 25330864 10.1007/s00381-014-2572-7

[17] Fujimoto A, Okanishi T (2022) Corpus callosotomy: editorial. Brain Sci 12:1006. 10.3390/brainsci12081006 36009068 10.3390/brainsci12081006 PMC9405958

[18] Harris WB, Brunette-Clement T, Wang A et al (2022) Long-term outcomes of pediatric epilepsy surgery: individual participant data and study level meta-analyses. Seizure 101:227–236. 10.1016/j.seizure.2022.08.010 36108556 10.1016/j.seizure.2022.08.010

